# Inflammatory biomarkers in type 2 diabetic patients: effect of glycemic control and impact of ldl subfraction phenotype

**DOI:** 10.1186/1475-2840-13-34

**Published:** 2014-02-04

**Authors:** Irene Vinagre, José Luis Sánchez-Quesada, Juan Sánchez-Hernández, David Santos, Jordi Ordoñez-Llanos, Alberto De Leiva, Antonio Pérez

**Affiliations:** 1 Endocrinology and Nutrition Department, Hospital de la Santa Creu i Sant Pau, C/ Mas Casanovas 90, Barcelona 08025, Spain; 2 Universitat Autònoma de Barcelona, Barcelona, Spain; 3 Cardiovascular Biochemistry, Biomedical Research Institute IIB Sant Pau, Barcelona, Spain; 4 CIBER of Diabetes and Metabolic Diseases (CIBERDEM), Barcelona, Spain; 5 Biochemistry Department, Hospital de la Santa Creu i Sant Pau, Barcelona, Spain; 6 Biochemistry and Molecular Biology Department, Universitat Autònoma de Barcelona, Barcelona, Spain; 7 CIBER of Biomedicine, Biotechnology and Nanomedicine (CIBERBBN), Barcelona, Spain

**Keywords:** Inflammatory biomarkers, C-reactive protein, Adiponectin, Monocyte chemotactic protein-1, Transforming growth factor-β_1_, Tumor growth factor β_1_, Type 2 diabetes, LDL phenotype, Atherogenic dyslipidemia

## Abstract

**Background:**

Type 2 diabetes mellitus (T2D) is associated with higher cardiovascular risk partly related to an increase in inflammatory parameters. The aim of this study was to determine the association of inflammatory biomarkers with low-density lipoprotein (LDL) subfraction phenotype and glycemic control in subjects with T2D and poor glycemic control.

**Methods:**

A cross-sectional study was performed comparing 122 subjects with T2D (59 ± 11 years old, body mass index 30.2 ± 5.6 kg/m2) with 54 control subjects. Patients with T2D were classified according to their LDL subfraction phenotype and inflammatory biomarkers (C-reactive protein, Interleukin-6, Interleukin-8, Transforming growth factor β_1_, Monocyte chemotactic protein 1, Leptin, Adiponectin) were evaluated according to the degree of glycemic control, LDL phenotype and other clinical characteristics. Forty-two subjects with T2D were studied before and after 3 months of improving glycemic control by different strategies.

**Results:**

Patients with T2D had higher C-reactive protein (CRP) and monocyte chemotactic protein-1 (MCP1) levels and lower adiponectin concentration, compared to controls. T2D subjects with body mass index ≥ 30 kg/m2 had higher CRP levels (5.2 ± 4.8 mg/l vs 3.7 ± 4.3 mg/l; p < 0.05). The presence of LDL phenotype B was related to higher levels of transforming growth factor-β_1_ (TGF-β_1_) (53.92 ± 52.82 ng/l vs 31.35 ± 33.74 ng/l; p < 0.05) and lower levels of adiponectin (3663 ± 3044 ng/l vs 2723 ± 1776 ng/l; p < 0.05). The reduction of HbA1c from 9.5 ± 1.8% at baseline to 7.4 ± 0.8% was associated with a significant reduction of TGF-β_1_ (41.86 ± 32.84 ng/l vs 26.64 ± 26.91 ng/l; p = 0.02).

**Conclusions:**

Subjects with T2D, especially those with LDL phenotype B and obesity, have higher levels of inflammatory biomarkers. Improvement of glycemic control reduces TGF-β_1_ levels, which may contribute partly to its renoprotective role.

## Background

Type 2 diabetes mellitus is one of the most common diseases in occidental society and it is associated with a high cardiovascular risk, not only due to the classical factors but also to a chronic low-grade inflammation [1]. In this sense, epidemiological studies have demonstrated an increase in plasma levels of inflammatory markers such as CRP, IL-6 and TNF-α in patients with metabolic syndrome and also in those with clinically overt T2D [2,3]. Other molecules such as the transforming growth factor (or tumor growth factor) β_1_[4], MCP1 [5,6] or LP-PLA2 [7] also present increased concentrations in T2D subjects.

A genetic predisposition associated to excessive caloric intake and a lack of physical excercise can lead to obesity and central adiposity. Then, this may result in adipose tissue dysfunction, macrophage infiltration and a greater release of cytokines such as IL-6 and TNF-α. Chronically elevated levels of these biomarkers promote insulinresistance in skeletal muscle and endothelial dysfunction, as well as liberation of CRP from the liver [8]. Moreover, hyperglycaemia also induces IL-6 production from endothelium and macrophages, which might worsen insulin liberation and signaling cascades. This suggests that improving glycemic control might reduce the inflammatory response supporting the link between inflammation and glucose metabolic disturbance.

On the other hand, previous studies have demonstrated a significant correlation between CRP levels and features of the metabolic syndrome, including adiposity, hyperinsulinemia, insulin resistance, hypertriglyceridemia and low HDLc [9-11]. Interestingly, lifestyle and pharmacological interventions in T2D such as statins, angiotensin receptor blockers and glitazones have demonstrated a beneficial effect on markers of inflammation [12-15]. However, the correlation between inflammatory markers and glycemic control is significant in some studies [16,17] but not in others [18], and available information about the effect of an improvement of glycemic control on inflammatory markers is very scarce [19,20]. Moreover, the impact of the presence of the atherogenic LDL subfraction phenotype, very common in T2D, on inflammatory markers has not been previously analyzed in these subjects.

Therefore, the aim of this study was to determine the association of inflammatory biomarkers with glycemic control and LDL subfraction phenotype.

## Methods

### Patients

This report includes a cross-sectional study with 122 consecutive poor controlled T2D patients who attended an out-patient diabetic clinic between 2007 and 2008 to optimize glycemic control. Patients were selected on the basis of poor glycemic control (HbA1C > 8.5%). As a control group, we selected 54 normoglycemic and normolipemic healthy subjects, without chronic pharmacological medication. This cohort of patients had been studied previously [21,22]. Anthropometrical and clinical characteristics of patients and controls, which have been reported elsewhere [22], are shown in Table 1. Briefly, previous pharmacological hypoglycemic treatment consisted of diet only (22%), oral agents (21.1%), insulin plus oral agents (29.4%) and insulin alone (27.5%). Pharmacological hypolipidemic treatment included statins (25.4%), fibrates (4.1%), statins plus fibrates (2.5%) and statins plus ezetimibe (1.6%). Patients with acute or chronic infections, active inflammatory disease or treatment with anti-inflammatory drugs were excluded. Subjects with CRP higher than 20 mg/L were also excluded from the study. A longitudinal study was also performed in a subgroup of 42 subjects with T2D and poor glycemic control, defined as HbA1c > 8.5%, who were recruited consecutively from the diabetes clinic [21]. All patients were evaluated before and after 3 months of glycemic optimization in a clinical practice setting with different strategies, which included lifestyle counseling and intensification of pharmacological hypoglycemic therapy with oral agents (metformin or metformin plus sulphonylurea) in 6 patients, insulin therapy in 31 patients or both (insulin plus metformin or insulin plus metformin and sulphonylurea) in 5 patients [21]. Lipid-lowering and antihypertensive therapy was unchanged.

**Table 1:** Anthropometric and clinical characteristics of control subjects and patients with type 2 diabetes at baseline

	**Control group (N = 54)**	**Type 2 diabetes (N = 122)**
Age (years)	56 ± 14	59 ± 11
Diabetes duration (years)	-	10.61 ± 10.26
Chronic complications:	-	
● Retinopathy (%)		29.7
● Nephropathy (%)^a^		32.4
● Coronary Heart Disease (%)		9.9
Antihypertensive medication (%)	-	67.9
Cholesterol lowering medication (%)	-	33.6
Weight (kg)	69.3 ± 11.4	82.1 ± 18.8*
Waist (cm)	92 ± 11	105 ± 14*
Body mass index (Kg/m2)	25.9 ± 3.5	30.2 ± 5.6*

Data are expressed mean ± SD.

^a^Nephropathy defined as impaired renal function (eGFR < 60 ml/minut) or 2 or more urinary determinations of microalbuminuria ≥ 20 mg/l or ≥ 30 mg/g of creatinine.

*Statistically significant (p <0.05).

The study was performed in accordance with the principles of the Declaration of Helsinki and was approved by the Hospital’s ethics committee. All patients and controls signed informed consent.

### Laboratory analysis

Blood specimens were obtained after overnight fasting. HbA1c was measured by ion-exchange high-performance liquid chromatography (HPLC; variant II, Bio-Rad) with a reference range of 4.6-5.8%. LDL subfraction phenotype was determined by measuring LDL size, determined by non-denaturing polyacrylamide gradient (2–16%) gel electrophoresis, as described [23]. LDL subfraction phenotype B was defined by a predominant LDL diameter lower than 25.5 nm, while phenotype A subjects had a LDL diameter higher than 25.5 nm. C-reactive protein was measured by a highly-sensitive commercial method (hsCRP, Roche Diagnostics) in Hitachi 917 autoanalyzer. Interleukin 6, IL-8, MCP1, TGF-β_1_, leptin and adiponectin were measured by ELISA from Bender Medsystems (IL-6, IL-8, MCP1 and TGF-β_1_), or R&D (leptin and adiponectin).

### Statistical analysis

Statistical analysis was performed using SPSS version 15.0 (SPSS Inc.). Before statistical analysis, normal distribution and homogeneity of the variances were tested using Kolmogorov-Smirnov and Levène tests, respectively. Data that were not normally distributed were logarithmically transformed before analysis. Groups were compared using Student’s unpaired t test for parameters with normal distribution or Mann–Whitney test for parameters with non-normal distribution. The effect of glycemic optimization was analyzed using Student’s paired t test for parameters with normal distribution or Wilcoxon test for parameters with non-normal distribution. Correlations between parameters were analyzed using the Pearson R test for variables with normal distribution and the Spearman test for variables with non-normal distribution. Data are expressed as mean ± standard desviation. P < 0.05 was considered significant.

## Results

**Figure 1:**
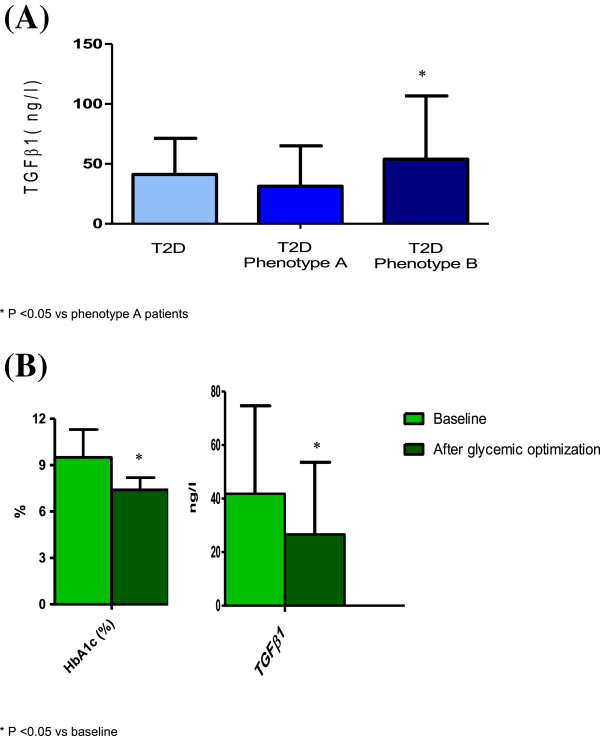
**TGFβ1 levels.** TGFβ1 levels in type 2 diabetic patients according LDL Phenotype **(A)**, and before and after glycemic control optimization **(B)**. T2D: type 2 diabetes.

Table 2 shows inflammation parameters of patients with T2D and controls. T2D subjects had higher CRP and MCP1 levels, and lower adiponectin concentrations than controls. One third (n = 44) of patients with T2D presented phenotype B, in contrast to the control group in which all subjects (n = 54) had phenotype A. Compared to T2D patients with LDL phenotype A, subjects with LDL phenotype B had higher concentrations of TGF-β_1_ (53.92 ± 52.82 ng/l vs 31.35 ± 33.74 ng/l; p < 0.05) and lower of adiponectin (3663 ± 3044 ng/l vs 2723 ± 1776 ng/l; p < 0.05) (Table 2, Figure 1A). T2D subjects with BMI ≥ 30 kg/m2 had higher CRP levels (5.2 ± 4.8 mg/l vs 3.7 ± 4.3 mg/l; p = 0.029), while those previously treated with metformin had lower levels of IL-6 (1.51 ± 3.86 ng/l vs 2.23 ± 4.4 ng/l; p = 0.046). There were no differences between patients treated with or without insulin and between those with or without lipid-lowering medication (data not shown). No correlation was found between glycemic control and inflammatory biomarkers.

**Table 2:** Inflammation parameters of control subjects and patients with type 2 diabetes mellitus, according LDL subfraction phenotype, at baseline: T2D: type 2 diabetes

	**T2D (N = 122)**	**T2D phenotype A (N = 77)**	**T2D phenotype B (N = 45)**	**Control group (N = 54)**
C-reactive protein (mg/l)	4.49 ± 4.67 ^a^	4.31 ± 4.52	4.86 ± 5.10	2.38 ± 3.30
Interleukin-6 (ng/l)	1.84 ± 3.99	2.24 ± 4.95	1.17 ± 0.83	1.25 ± 1.05
Interleukin-8 (ng/l)	15.91 ± 12.85	17.46 ± 13.74	13.20 ± 10.87	12.58 ± 9.29
Transforming growth factor β1 (ng/l)	41.19 ± 30.08	31.35 ± 33.74	53.92 ± 52.82 ^b^	39.55 ± 42.86
Monocyte chemotactic protein 1 (ng/l)	198.0 ± 132.9 ^a^	190.6 ± 125.6	212.1 ± 146.21	154.3 ± 83.0
Leptin (ng/l)	8980 ± 8194	9066 ± 8786	8787 ± 7184	7819 ± 5866
Adiponectin (ng/l)	3272.0 ± 2671 ^a^	3663 ± 3044	2723 ± 1776 ^b^	5369 ± 3297

Data are expressed mean ± SD.

^a^, P <0.05 vs control group; ^b^, P <0.05 vs phenotype A patients.

Table 3 and Figure 1B show clinical characteristics and inflammatory biomarkers of a subgroup of T2D patients before and after glycemic optimization. After a follow-up of 3 months, HbA1c decreased from 9.5 ± 1.8% to 7.4 ± 0.8%, without changes in body weight. Improvement of glycemic control was associated with a significant reduction of TGF-β_1_ (41.86 ± 32.84 ng/l vs 26.64 ± 26.91 ng/l; p = 0.02). However, changes in TGF-β_1_ levels did not correlate with those observed in HbA1c.

**Table 3:** Clinical characteristics and inflammation parameters of patients with type 2 diabetes mellitus before and after glycemic optimization (n = 42)

	**Baseline**	**After glycemic optimization**
Male/female	27/15	-
Age (years)	61 ± 12	-
Diabetes duration (years)	37 ± 14	-
Chronic complications:		
● Retinopathy (%)	59	-
● Nephropathy (%)	51.3	-
● Coronary Heart Disease (%)	17.9	-
Insulin use (%)	75	85.7
Cholesterol lowering medication (%)	59.5	59.5
Weight (kg)	80.35 ± 19.52	80.02 ± 19.71
Body mass index (Kg/m2)	29.0 ± 5.8	28.8 ± 6
HbA1c (%)	9.5 ± 1.8	7.4 ± 0.8*
C-reactive protein (mg/l)	3.20 ± 2.86	3.15 ± 3.26
Interleukin-6 (ng/l)	1.49 ± 1.12	1.29 ± 0.70
Interleukin-8 (ng/l)	12.84 ± 7.57	13.82 ± 10.35
Transforming growth factor β1 (ng/l)	41.86 ± 32.84	26.64 ± 26.91*
Monocyte chemotactic protein 1 (ng/l)	224.31 ± 76.03	227.48 ± 132.79
Leptin (ng/l)	7594 ± 8852	7817 ± 7295
Adiponectin (ng/l)	3673 ± 3455	4084 ± 3927

Data are expressed mean ± SD.

*p <0.05 vs baseline.

## Discussion

The present study confirms the low grade systemic inflammation status in T2D patients which is related to features of the metabolic syndrome. However, the main and novel findings are related to increased levels of TGF-β_1_ concentration in patients with LDL phenotype B, compared to those with phenotype A, and its reduction after a marked improvement of glycemic control. These findings suggest that TGF-β_1_ may be a link to glycemic control and atherogenic dyslipidemia with the development of diabetic nephropathy.

### Systemic inflammation status in T2D subjects

The analysis of inflammatory parameters showed that plasma concentrations of CRP and MCP1 were higher and that of adiponectin was lower in T2D patients than in control subjects. This observation indicates a greater global systemic inflammation status in diabetic patients, which has been previously suggested that may reflect the activity of the underlying atherosclerotic process [24], and it is concordant with several cross-sectional studies which have shown an increase of CRP levels in patients with diabetes [25-27]. Although the results reported in the literature on the association between IL-6 and T2D are contradictory [26,28,29], the absence of statistical differences in IL-6 and other inflammatory parameters could be related to the fact that most patients were treated with medications such as metformin, antihypertensive agents and lipid-lowering drugs, which have demonstrated a beneficial effect on inflammation [12-14]. In addition, the available information about the relationship between inflammatory biomarkers and the characteristics of the diabetes is limited. Some studies have demonstrated significant correlation between CRP levels with features of the metabolic syndrome, including adiposity, hyperinsulinemia, insulin resistance, hypertriglyceridemia and low HDLc [9,30]. The preponderance of small LDL particles (LDL phenotype B) is a component of atherogenic dyslipidemia. In accordance, we have observed that obese T2D subjects have higher CRP concentrations and inflammatory status was more unfavorable in patients with LDL phenotype B, who had higher concentration of TGF-β_1_ and lower of adiponectin than patients with phenotype A. To our knowledge, no other studies have examined this association, but there are some publications agreeing that serum levels of TGF-β_1_ are higher in patients with metabolic syndrome than those without it [31] and that oxidized low-density lipoprotein, which is increased in subjects with LDL phenotype B, is associated with TGF-β_1_ in T2D subjects [32]. Finally, TGFβ family members have been involved in vascular remodeling defects in experimental models [33], which could contribute to explain the worse prognostic of atherosclerotic cardiovascular disease in diabetic patients.

### The effect of improving glycemic control on markers of inflammation in T2D

It has been shown that in the individuals with impaired glucose tolerance [34] the low-grade chronic inflammation is related to glucose metabolic disturbance and a growing body of evidence supports the hypothesis that chronic systemic inflammation contributes to decrease insulin sensitivity [35]. Moreover, hyperglycemia is a significant stressor that has also been shown to cause chronic inflammation [36]. However, the relationship between inflammatory markers and glycemic control is still not fully understood. In our study, no inflammation marker correlated with HbA1c levels. These results are consistent with the findings of previous cross-sectional studies which have found an inconsistent association between inflammation and blood glucose levels [16-18], possibly due to previous treatment with metformin, statins and antihypertensive agents, which may limit the power to detect an association between HbA1c and inflammatory markers. In the longitudinal study, we demonstrated for the first time that improvement of glycemic control by different therapeutic strategies can lower TGF-β_1_ concentrations. These results are consistent with the findings in diabetic rats treated with insulin and with the increased expression of TGF-β_1_ receptor in the diabetic kidney and in mesangial cells cultured in high glucose [37,38]. The lack of significant correlation between the magnitude of the reductions in HbA1c and TGF-β_1_ after glycemic optimization may reflect differences in the relative importance of the local versus systemic blood glucose levels, and it does not preclude an underlying relationship between these two parameters. The lack of change in CRP and other inflammatory markers in our study cannot be attributed to weight gain or pharmacological therapy, and confirms the results in the few studies which have evaluated the effect of improving glycemic control on markers of inflammation in type 1 diabetes (DCCT) and T2D [19,20]. In the DCCT study, intensive glycemic control was not associated with changes in levels of CRP and among intensively treated subjects who gained the most weight, there was a significant rise. In T2D, in a study of 18 patients who had been hospitalized to initiate insulin therapy because of poor diabetes control, CRP values, but not MCP1 and fibrinogen levels, were decreased 2 weeks after initiation of insulin therapy. This effect could result from anti-inflammatory effects of insulin [18], although these findings were not confirmed in the study of Pradhan et al. [19]. They found that, in patients with recent-onset T2D, treatment during 14 weeks with insulin glargine or metformin compared with placebo did not reduce CRP, IL-6 and soluble tumor necrosis factor receptor 2, despite improving glucose control. In the present study, the duration of the study was sufficient on the basis of assessment of glycemic control by HbA1c, and a marked improvement of glycemic control was obtained, being nearly normal in most of the patients without changes in body weight. Furthermore, lipid-lowering and antihypertensive therapy was unchanged and in most patients insulin therapy was the strategy used to improve glycemic control.

### The possible role of TGF-β_1_ in diabetic nephropathy

The critical role of hyperglycemia in the genesis of diabetic nephropathy has been established by cell culture studies, experimental animal models, and clinical trials. Certain cytokines have been identified as likely mediators of the effects of high ambient glucose on the kidney. TGF-β_1_, a known pro-fibrotic factor, activates the production of extracellular matrix by mesangial cells and interstitial fibroblasts in the kidneys, and thus contributes to the manifestation of diabetic kidney disease through a number of key pathological events leading to reduced glomerular filtration and impaired renal function [39,40]. In mesangial cells, it has been demonstrated that the expression and activity of TGF-β_1_ are directly influenced by glucose levels [38]. Moreover, atherogenic dyslipidemia is associated with the onset and progression of nephropathy in T2D subjects, by a mechanism not fully understood [41,42]. Therefore, the finding of a higher concentration of TGF-β_1_ in patients with atherogenic dyslipidemia provides a potential explanation.

In conclusion, the present study confirms that low-grade chronic inflammation in T2D is related to LDL phenotype B and obesity. In addition, our data demonstrates that improvement of glycemic control reduces TGF-β_1_ levels, through an unknown mechanism. These findings might support the role of TGF-β_1_ as a likely mediator of the effects of high ambient glucose on the kidney and the beneficial effects of strict glucose control on the development of diabetic nephropathy. However, future studies in larger groups of patients should be performed to confirm these findings.

## Abbreviations

T2D: Type 2 diabetes mellitus; CRP: C-reactive protein; IL-6: Interleukin-6; TNF: Tumor necrosis factor; MCP1: Chemotactic monocyte protein 1; LP-PLA2: Lipoprotein-associated phospholipase A2; LDLc: Low density lipoproteins cholesterol; HDLc: High density lipoproteins cholesterol; HbA1C: Glycated haemoglobin; TGF-β1: Transforming growth factor or tumor growth factor β_1_; BMI: Body mass index.

## Competing interests

The authors declare that they have no competing interests.

## Authors’ contributions

IV selected patients, performed statistical analysis and wrote the manuscript. JLS-Q conceived of the study, researched data and reviewed the manuscript. JS-H performed statistical analysis and reviewed the manuscript. DS researched data. JO-L participated in the design of the study, contributed to the discussion and reviewed/edited the manuscript. ADL participated in the design of the study, contributed to the discussion and reviewed/edited the manuscript. AP conceived of the study, selected patients and wrote the manuscript. All authors read and approved the final manuscript.
